# Synthesis of Programmable Bioelectronic Yeast for Biohybrid Futures

**DOI:** 10.1049/enb2.70007

**Published:** 2026-05-11

**Authors:** Paige E. Erpf, Bill D. Kinder, Edward Archer, Roy S. K. Walker, Isak S. Pretorius, Ian T. Paulsen

**Affiliations:** ^1^ Australian Research Council Centre of Excellence in Synthetic Biology Macquarie University Sydney New South Wales Australia; ^2^ The Chancellery Macquarie University Sydney New South Wales Australia; ^3^ The Australian Genome Foundry Sydney New South Wales Australia

**Keywords:** bio‐design, genetic engineering, microbial engineering, Sc2.0 chromosomes, synthetic biology

## Abstract

The budding yeast *Saccharomyces cerevisiae* has moved beyond an evolved and domesticated single‐celled microorganism into a deliberately engineered biological substrate. Advances in synthetic genomics, illustrated by the international Synthetic Yeast Genome (Sc2.0) project, have reframed the yeast genome as a designable and programmable system. This article examines how locus standardisation, genome refactoring, controlled genomic plasticity and orthogonal regulatory systems collectively establish yeast as a programmable platform. Yeast is then viewed as an analogue of electronic systems in which genetic circuits, memory and population‐level computation are compared to logic gates, storage and distributed system architectures. These capabilities position yeast to move beyond conventional metabolic engineering towards hybrid systems that integrate biological information processing with electronic and computational components. Achieving such integration requires careful consideration of the interfaces between biological and electronic domains, including how biological states can be coupled to electronic systems through electrochemical, chemical, optical and mechanical transduction, and how electronic inputs can be delivered in forms that can be recognised and processed by engineered cells. Finally, both the principal bottlenecks and key enabling advances are discussed, highlighting how recent developments suggest that synthetic yeast is approaching readiness as a foundational platform for bioelectronic and hybrid living systems.

## Graphical abstract

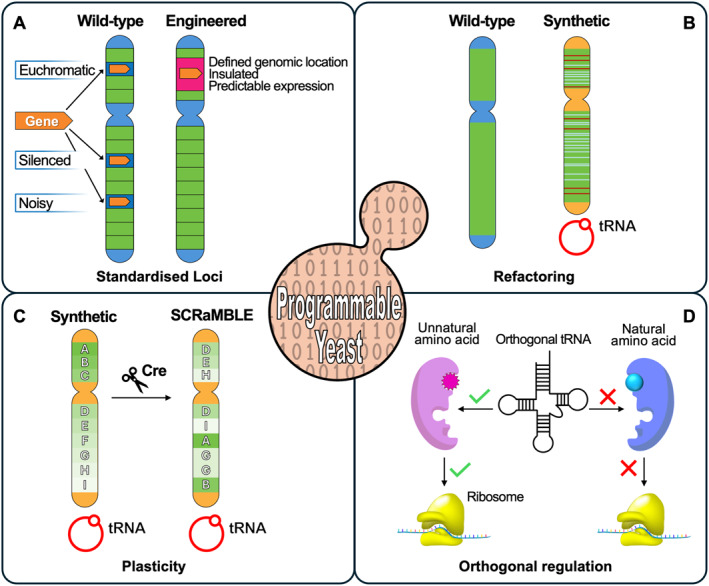

## 1 Yeast: From an Evolved Organism to an Engineered Biological Substrate

Baker's yeast, *Saccharomyces cerevisiae*, is the product of a long evolutionary history shaped by both natural and artificial selection. Emerging ∼100 million years ago, *Saccharomyces* species evolved as competitive specialists in sugar‐rich environments, acquiring the capacity to produce, tolerate and metabolise ethanol [1].

The relationship between yeast and humans grew during the Neolithic period, when the adoption of agriculture created new, stable fermentation environments [2, 3, 4]. Over millennia, repeated domestication events further shaped *Saccharomyces* genomes, favouring traits associated with fermentation efficiency, robustness and sensory outcomes [5]. In this sense, yeast represents one of the earliest examples of a microbial biological system shaped not only by natural selection but also by sustained artificial selection.

The transition from domestication to deliberate biological engineering began in the twentieth century, when yeast emerged as a foundational eukaryotic model organism [6]. Its short generation time, ease of cultivation, stable haploid and diploid life cycles and compatibility with recombinant DNA technologies made *S. cerevisiae* uniquely amenable to genetic analysis and manipulation.

Despite these advantageous traits, early engineering efforts were hindered by the limited knowledge of the complete genetic code. The completion of the first eukaryotic genome sequence in 1996 marked a critical turning point, transforming yeast from a genetically tractable organism into a genomically defined system [7]. Subsequent advances in functional genomics [8, 9], systems biology [10] and metabolic engineering [11] further consolidated yeast's role as both an experimental model and an industrial chassis, supporting applications spanning basic research, biotechnology and manufacturing.

Only recently, through the advent of synthetic biology and synthetic genomics, has the application of rational engineering principles begun to unlock the black box of the yeast genome; introducing the view that yeast is an engineerable system that combines the concepts of biology with rational design. Advances in DNA synthesis and synthetic biology have introduced new tools and approaches that have enabled the design, construction and testing of biological systems.

These rational engineering principles, including standardisation, modularity, orthogonality and hierarchy of abstraction [12], together with the iterative design–build–test–learn cycle (DBTL), motivate the treatment of biological systems as programmable substrates rather than a collection of isolated genetic modifications. Although such principles have recently permitted new forms of interaction between biological and digital systems, their successful application depends on the existence of biological platforms that can reliably support abstraction, composition and control. This review explores these principles in a biological context and examines how yeast has been re‐engineered to function as a programmable system. It then considers how such systems can be interfaced with electronic hardware to enable integrated bioelectronic control, presenting a testbed and pathway for future biohybrid devices.

## 2 Synthetic Yeast as a Programmable System

Early efforts in yeast engineering focused on local genetic modifications, rather than deliberate genome‐scale design. It was not until the early 2010s that the synthetic yeast genome project (Sc2.0) represented a fundamental departure from earlier modes of genetic engineering [13]. Conceived as an international effort to chemically synthesise and rationally redesign the entire *S. cerevisiae* genome, Sc2.0 reframed the genome itself as an explicitly designable substrate, capable of supporting genome‐scale design principles and a programmable genetic code [14].

Through the introduction of systematic changes to the synthetic genetic code, including streamlined genomic organisation, modularisation and LoxPsym sites for controlled rearrangement, Sc2.0 began to separate the cell's potential from standard evolutionary rules [14]. Importantly, this process was not linear. The construction of synthetic chromosomes revealed unanticipated dependencies, fitness defects and design trade‐offs, necessitating iterative debugging and refinement [15]. These corrective cycles yielded critical and often unexpected insights into gene function and genome organisation, demonstrating that genome scale design is inherently an engineering process shaped by empirical feedback from living biological systems, rather than a purely computational specification.

Following the completion of all 16 synthetic chromosomes, the yeast genome can no longer be regarded solely as an organism to be modified at the individual gene level. Instead, it constitutes a programmable system whose structure, dynamics and evolutionary potential can be deliberately specified, tested and revised. The lessons emerging from Sc2.0 extend beyond yeast itself, providing important knowledge and principles for future synthetic genomes [16, 17] and underscores the importance of DBTL cycles in large‐scale biological engineering.

This shift from mere modification towards reshaping the native genome completely establishes a foundation for treating yeast as an engineered and programmable substrate. This allows for biological systems to be designed not only for biochemical function, but also for higher‐order information processing and integration with external computational and electronic systems. For a high‐level perspective on this emerging technological shift, see Pretorius et al. [13] and references therein.

### 2.1 Standardised Loci for Sensing, Computation and Output

A defining feature of programmable systems is the ability to connect defined inputs to predictable outputs through reusable modules. In engineering, this is enabled by standardisation, which restricts how components interact and allows complex systems to be assembled from well‐characterised parts. In a biological context, this requirement is complicated by the fact that gene expression is strongly influenced by the genomic landscape, with chromosomal position [18], local chromatin state and neighbouring regulatory elements all contributing to variability [19]. In yeast, these positions have been recognised as a source of noise, irreproducibility and instability, leading to efforts that separate genetic functions from the local genomic context [20, 21].

To address these biological challenges, standardised genomic loci have been developed that support reproducible integration and expression of heterologous genetic elements into yeast. Early strategies focused on integration within coding loci commonly used as selectable markers, including *URA3*, *LEU2* and *HIS3*, whereas later approaches identified intergenic regions suitable for stable gene insertion [22, 23, 24]. More recently, systematic screening efforts evaluated 125 potential integration sites across the yeast genome and identified 76 loci that supported efficient integration and stable gene expression without impairing host fitness [23].

**FIGURE 1:**
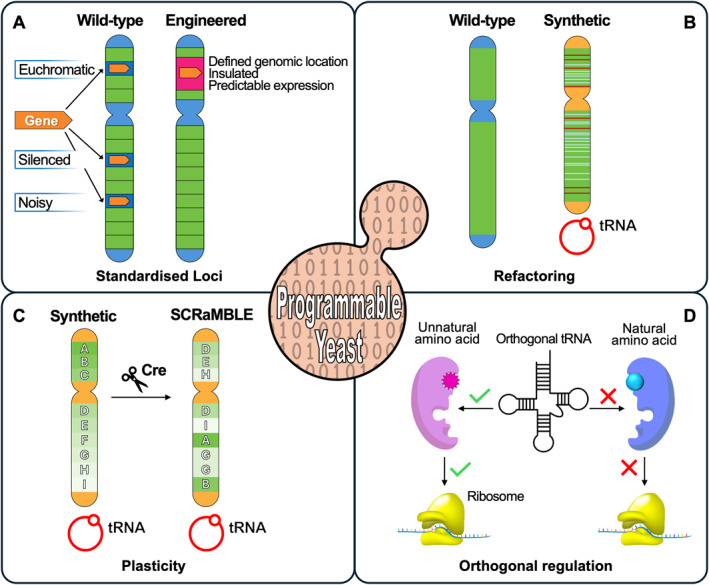
Synthetic yeast as a programmable system. (A) Standardised genomic loci. The wild‐type chromosome contains integration sites in euchromatic, silenced and transcriptionally noisy regions, leading to variable gene expression. In contrast, the engineered chromosome contains a defined, insulated loci that supports predictable and stable expression. (B) Genome refactoring. The wild‐type chromosome is compared with a synthetic chromosome in which repetitive elements (including native tRNA genes) have been removed, stop codons have been reassigned (red lines) and loxPsym sites (blue lines) have been introduced. (C) Controlled genomic plasticity. A synthetic chromosome containing loxPsym sites at defined positions undergoes Cre recombinase‐mediated rearrangement, generating alternative genomic configurations through SCRaMbLE. (D) Orthogonal regulation using engineered tRNAs. An orthogonal tRNA–aminoacyl‐tRNA synthetase pair selectively incorporates unnatural amino acids during translation while remaining insulated from the native translational machinery.

By constraining where sensing, processing or output modules are encoded, standardised loci act as interfaces within the genome, enabling genetic elements to be designed, tested and reused independently of chromosomal context. In addition, integration at standardised loci reduces variability arising from position effects, resulting in more stable expression levels and lower noise across cells and experiments (Figure 1A). This approach adopts principles used in conventional engineering disciplines, where standardised interfaces reduce unintended effects and improve system predictability. Although biological computation differs fundamentally from digital computation, the use of standardised loci enables a more consistent implementation of genetic logic operations and cellular responses that depend on the regulatory state across strains and applications.

In programmable yeast, standardised loci provide a common ground in which sensing, computation and output can be systematically organised, improving comparability across designs while reducing the need for locus‐specific optimisation. By constraining genetic functions to well‐defined regions in the genome, such loci promote more consistent gene expression, integration rates and responses across strains and experimental conditions. Although encoding these functions at defined loci does not eliminate biological variability, it greatly stabilises engineered behaviour and facilitates reuse and overall refinement of otherwise complex biological systems.

### 2.2 Genome Refactoring and Modularity

In engineering disciplines, refactoring often refers to the restructuring of a system to improve its organisation, readability and adaptability without altering core function. When this concept is applied to biological systems, it provides a useful lens to better understand the effects of genome‐scale redesign, where gene function is largely preserved while the underlying genomic architecture is deliberately reorganised for improved engineering strategies.

Genome refactoring in Sc2.0 involved a series of design choices aimed at reducing structural complexity and increasing behavioural control while maintaining cell fitness. These changes included the removal of introns and repetitive elements, the recoding of stop codons to enable future expansion of the genetic code and the introduction of sequence watermarking (PCRtags) to distinguish synthetic DNA from native DNA [14] (Figure 1B). Collectively, these modifications do not fundamentally alter yeast biology but better help to define biological function from an engineering perspective.

A particularly illustrative example of this refactoring is the consolidation of all tRNA genes onto a dedicated and de novo designed synthetic chromosome (neochromosome) [25]. By relocating these essential elements from their native loci, the function of these translational molecules is retained while improving the overall stability of the synthetic genome. This separation transforms tRNA expression from a genomic constraint into a module isolated from the rest of the genome.

Genome refactoring enables modularity at both architectural and functional levels, reflecting different ways in which biological systems can be partitioned, reorganised and reused. At the architectural level, the Sc2.0 framework introduces an embedded form of in‐built modularity through SCRaMbLE (synthetic chromosome rearrangement and modification by LoxP‐mediated evolution) [26]. SCRaMbLE incorporates recombination sites at defined genomic locations which then enable inducible and constrained rearrangements of synthetic chromosomes (Figure 1C). This plasticity is conditional: recombination events occur only following activation and are limited to predefined regions. However, SCRaMbLE frequently generates inviable or severely fitness‐compromised genotypes, necessitating robust selection or screening to recover viable variants. As a result, exploration of new genomic configurations is inherently coupled to viability.

At the functional level, modularisation is the reorganising of metabolic or regulatory pathways into discrete, interchangeable units that are insulated from the rest of the system. In yeast, this principle has been demonstrated through pathway‐swapping approaches that consolidate the glycolytic pathway into a single genomic locus, enabling systematic replacement and combinatorial reconfiguration [27]. However, these implementations remain largely pathway‐specific and have not yet been extended into a systematic, genome‐wide design framework comparable to the architectural refactoring achieved in Sc2.0.

### 2.3 Orthogonal Regulatory Systems

Programmable systems require the ability to coordinate functions without unintentionally interfering with core system components. In engineering, this is achieved by separating control logic from the processes it governs, allowing control signals to be composed, modified and reused without direct entanglement with internal system dynamics. In biological systems, gene regulation is highly interconnected, with transcriptional and post‐transcriptional processes embedded within dense endogenous regulatory networks shaped by chromatin state, regulators and feedback mechanisms.

Orthogonal regulatory systems address this challenge by introducing new levers of control that are functionally insulated from native gene regulation. In yeast, such systems have been implemented using transcriptional and genetic‐level control mechanisms, including engineered transcription factors [28], synthetic promoters [29, 30], recombinase‐based logic [31, 32], programmable DNA‐binding proteins [33] and unnatural amino acids using engineered tRNA–aminoacyl‐tRNA synthetase pairs that do not cross‐react with endogenous translational machinery [34, 35] (Figure 1D), as well as post‐translational control strategies such as chemically induced dimerisation [36] and split intein‐based systems [37] that enable rapid modulation of protein activity. By minimising crosstalk with endogenous regulators across complex gene expression networks, orthogonal systems enable engineered circuits to respond to defined inputs with reduced sensitivity to cellular state or environmental fluctuations.

In programmable yeast, orthogonal regulatory systems provide a critical mechanism to better control cell behaviour by enabling defined regulatory pathways to be designed, refactored and introduced within a standardised genome. Compared to defined electrical systems, orthogonality in biological systems is relative to other components of cell behaviour rather than absolute. Engineered biological systems will continue to operate within a shared biochemical environment and remain subject to resource competition, metabolic burden and physiological constraints. Nevertheless, even partial insulation can substantially improve system behaviour and support the construction of complex behaviours from simpler components.

## 3 Biological Computation and Information

On a fundamental level, these advances in designing a programmable yeast enable the host strain to be treated not merely as a modified cell factory but as a higher‐order system capable of biological information processing. Within this context, biological computation refers not to true digital calculation in a classical sense but to the capacity of engineered genetic systems to sense, integrate, store and act upon information in a predictable manner. Analogous to electronic systems, these capabilities are realised through genetic circuits that implement logic operations, memory elements that enable state retention and population‐level control that distribute computation across interacting cells. Together, these constructs define the functional repertoire through which programmable yeast can implement computation‐like behaviours.

Before discussing biological computation in detail, it is important to clarify the limits of the analogy between engineered biological systems and electronic computation. Biological computation does not operate through deterministic, binary switching in the same way as digital electronic circuits. Instead, cellular systems function within a biochemical environment governed by stochastic molecular interactions, continuous signal levels and significant variability across cells and populations. Biological processes are inherently noisy, often reversible and typically operate through parallel biochemical reactions rather than sequential logic operations. They also differ fundamentally in their energetic basis, relying on distributed metabolic processes rather than discrete electrical power and clocked architectures. Despite these differences, the conceptual framework of computation remains useful as a design heuristic. Describing genetic circuits, memory and population‐level coordination in computational terms provides a structured language for reasoning about how biological systems sense, process, store and respond to information, while acknowledging that the underlying mechanisms differ substantially from those of conventional electronic systems.

### 3.1 Genetic Circuits

**FIGURE 2:**
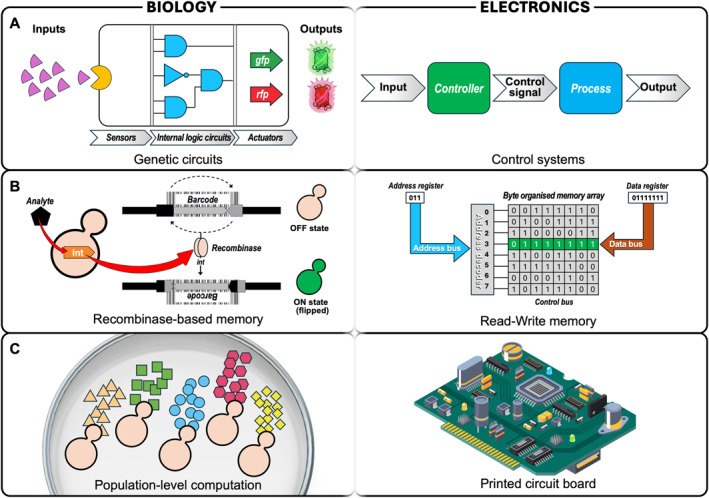
Analogies between biological and electronic information processing. (A) Genetic circuits in yeast integrate molecular inputs through regulatory logic and actuators to generate defined outputs, analogous to electronic control systems that process inputs through controllers and controlled processes. (B) Recombinase‐based genetic memory encodes information through site‐specific DNA rearrangements, providing stable, heritable state storage analogous to electronic read–write memory. (C) Population‐level computation, in which specialised subpopulations perform distinct sensing, processing or output functions, is analogous to the organisation of electronic components on a printed circuit board, where discrete elements cooperate to implement complex system behaviour.

Genetic circuits enable logical control within living cells by linking defined molecular inputs to predictable outputs. Early work in synthetic biology demonstrated that gene regulatory elements could be arranged to perform functions analogous to logic gates, establishing that biological regulation can be divided into abstract functional units with well‐defined input–output relationships [38, 39] (Figure 2A). Building on this foundation, individual logic elements can be combined to form genetic circuits capable of more complex behaviours, including the integration of multiple regulatory inputs to implement multi‐input decision‐making [40], feedback structures that stabilise or tune dynamic responses [41], and conditional programmes that activate distinct outputs depending on system state [42]. Although biological systems differ fundamentally from electronic circuits, implementing these circuit‐level abstractions has proven valuable to help with regulatory complexity that guide rational design.

In yeast, the development of genetic circuits has benefited from the organism's genetic tractability and significant knowledge of eukaryotic regulatory machinery [43]. Orthogonal regulatory components, including engineered promoters [44], transcription factors [45] and DNA‐binding proteins [46] have been used to construct circuits implementing Boolean logic [47], analogue signal processing and dynamic regulatory behaviours. Importantly, these circuits do not replace endogenous regulation, but are layered upon it, highlighting both the opportunities and constraints associated with computation in living systems.

For programmable yeast, genetic circuits form a foundational layer of biological computation, which will enable information to be encoded, processed and transmitted through engineered regulatory interactions. Notably, these circuit‐level capabilities underpin more advanced functions such as memory and population‐level computation, which build upon and extend the same abstraction principles.

### 3.2 Genetic Memory: Recombinase and Epigenetic Marks

Although genetic circuits enable the processing of information, many engineered behaviours require the ability to retain information about past events. In engineering systems, this corresponds to memory elements that store the system state beyond the duration of an input signal. In biological systems, memory is defined by a persistent alteration to the cell state following the removal of an initiating signal. For programmable biological platforms, memory enables history‐dependent behaviour and coordination across timescales that exceed those of transient gene expression (Figure 2B).

Engineered memory systems in yeast include two major classes, namely recombinase‐based [48, 49] and epigenetic [50]. Recombinase‐based systems encode information directly into DNA through site‐specific rearrangements that are irreversible or conditionally reversible. By converting transient inputs into stable genomic states, these systems function as digital memory elements that can record the occurrence, and in some designs, the order of specific signals. Because the encoded state is stored in DNA, recombinase‐based memory is more persistent, providing a record of past inputs with minimal ongoing regulatory activity.

In contrast, epigenetic memory arises from chromatin‐mediated regulation rather than sequence alteration. In yeast, histone modifications, nucleosome positioning and chromatin‐associated feedback can generate long‐lived transcriptional states that persist through multiple generations [51]. These mechanisms function as analogue or semi‐stable memory elements, encoding prior regulatory or environmental conditions while retaining reversibility and flexibility. Although epigenetic memory is more sensitive to cellular context, it enables adaptive and reversible behaviours that are difficult to achieve with permanent genomic rearrangements.

Recombinase‐based and epigenetic memory systems extend circuit‐level computation by supporting persistent and multi‐layered information storage in living cells. In programmable yeast, the coexistence of these genetic memories can provide complementary modes of information retention, analogous to persistent and volatile memory in engineered systems. These capabilities will enable information transfer across cell lineages and are likely to be essential for applications requiring sustained function, environmental responsiveness or coordination with external control systems.

### 3.3 Population‐Level Computation

As the complexity of engineered genetic systems increases, implementing all computational functions within a single cell can impose substantial design constraint and burden on the host cell [52]. Genetic circuits compete for shared cellular resources, and the accumulation of sensing, logic and memory modules can introduce unintended interactions coupling, reduced robustness and metabolic burden. In engineering terms, these limitations motivate distributed architectures in which computation is partitioned across interacting units rather than concentrated within a single system.

In population‐level computation, distinct cells or subpopulations can be engineered to perform specialised functions such as sensing, processing, memory or output generation (Figure 2C). By partitioning tasks across a consortium, individual cells maintain simpler internal genome architectures while collectively performing behaviours that exceed what is feasible within a single cell. This division of labour is functionally analogous to distributed computing architectures in which complex behaviour emerges from coordinated interactions among specialised components [53, 54]. Control‐engineering‐based approaches may be used to address practical challenges, including the competitive exclusion principle in complex environments, such as in the case of wine fermentation [55].

Cell‐to‐cell communication provides the coordination that enables distributed biological computation. In microbial systems, quorum sensing has been widely adopted as a programmable communication mechanism, allowing cells to exchange information about the population state and coordinate gene expression in response to shared signals [56]. In yeast, the native pheromone‐based mating signalling (α‐factor/a‐factor) has been extensively engineered to enable synthetic cell–cell communication and coordination [57]. It can also be further extended through mating‐type switching to dynamically reconfigure population behaviour [58]. Such systems enable coordinated behaviours and signal integration across populations, offering advantages in terms of chassis compatibility and reduced crosstalk compared to heterologous quorum sensing circuits. This allows for the extension of computation beyond the intracellular scale to the level of populations and consortia.

Population‐level computation can also incorporate memory at the level of lineages or communities. Heritable genetic or epigenetic states can propagate through subpopulations, enabling temporal integration and long‐term information storage that persists beyond individual cells. Such population‐encoded memory further expands the computational potential of engineered biological systems.

In programmable yeast, population‐level strategies can offer a practical route to scaling computational complexity while preserving robustness and tractability. By distributing information processing across interacting cells and coupling it through defined communication channels, engineered yeast populations can support sophisticated, resilient behaviours that are well suited to long‐term operation and integration with external control and bioelectronic systems.

## 4 Interfaces: Where Biology Meets Electronics

As engineering and biological approaches converge, the distinction between the biological and digital worlds becomes increasingly blurred. When programmable yeast is viewed as an information processing system, analogous in function (although not in mechanism) to an electronic chip, a central question becomes how such a biological system can be coupled to electronic hardware. Answering this question requires a clear understanding of bioelectronics and the nature of the interface between living cells and electronic systems [59].

Bioelectronics is broadly defined as the branch of science concerned with either the application of biological materials and processes in electronic devices, or the use of electronic systems to sense, control and interact with living systems. This review focuses on the latter, namely the integration of electronic systems with engineered yeast as dynamic, programmable organisms capable of generating, storing and responding to information. In this context, a bioelectronic interface defines how information crosses the boundary between biology and electronics. Information transfer can occur from biological systems to electronic devices, either through (i) the conversion of cellular states into measurable electronic signals (biological‐to‐electronic transduction) or (ii) from electronic systems to biology via external control inputs that influence cellular behaviour (electronic‐to‐biological control). Both of these interactions can consequently be viewed as being either unidirectional, forming open‐loop systems in which biology and electronics act independently, or bidirectional, where electronic measurement and control are linked through closed‐loop feedback systems.

### 4.1 Biological‐to‐Electronic transduction

Biological‐to‐electronic transduction refers to the conversion of internal biological states into signals that can be detected, interpreted and acted upon by electronic systems. In classical biology, transduction typically describes the relay of signals within cells, for example, from extracellular cues to intracellular responses. In bioelectronic systems, the term instead signifies the integration of biological activity or signals that are compatible with electronic measurement and control.

Several distinct transduction modalities have been explored (Table 1) to link internal biological states to electronic systems, including electrochemical (redox‐based; [60, 61], chemical [62], optical [63] and physical or mechanical approaches [64]). Table 1 summarises these modalities across a range of biological systems, highlighting both the diversity of available approaches and the limited number currently demonstrated in *S. cerevisiae*. Below, each approach is briefly discussed, highlighting how biological information is extracted, the extent to which measurement perturbs cellular physiology and its compatibility with scalable, living platforms such as yeast.

**TABLE 1:** Biological‐to‐electronic transduction modalities and representative signals.

	Species	What was detected	Technology	References
Electrochemical	*Saccharomyces cerevisiae*	Ferri/ferrocyanide	Linear sweep voltammogram (LSV)	[56]
	*S. cerevisiae*	Fe(CN)64−/Fe(CN)63−	Glassy carbon electrode (GCE), Nafion‐dispersed oxidised multi‐walled carbon nanotubes (OMWCNT)	[57]
	*Escherichia coli*	LacZa reporter protein	Electrochemical microfluidic chip	[58]
	*Bacillus subtilis*	DHA/AQ	Silicon nanowire field‐effect transistors (SiNW FET)	[59]
	*Pseudomonas aeruginosa*	Electric current	Microbial fuel cell	[60]
Chemical	*S. cerevisiae*	Glycolytic flux	Flow cytometry and microscopy	[61]
	*S. cerevisiae*	Ethanol	Conductometric computer‐controlled sensor	[62]
	*S. cerevisiae*	Ethanol	Direct catalytic fuel cell	[63]
	*E. coli*	O_2_ conc. and pH	Microfluidics	[64]
	*E. coli*	Indole and graphene	Graphene field‐effect transistors (G‐FET)	[65]
	*P. aeruginosa*	Proteolytic activity	Magnetic nanoparticles	[66]
	*Bacillus megaterium*	Arsenic	Complementary metal‐oxide‐semiconductor (CMOS)	[67]
Optical	*S. cerevisiae*	NADH fluorescence	2D fluorescence spectroscopy	[68]
	*S. cerevisiae*	eGFP	4‐chamber microfluidic	[69]
	*E. coli*	luxCDABE	Optoelectronic circuits	[70]
	*E. coli*	Cerulean CFP	Microscope‐coupled LCD projector	[71]
	*Pyrocystis lunula*	Luciferin	CMOS camera + microscope	[72]
Mechanical	*S. cerevisiae*	Cell wall nano‐motions	Atomic force microscopy (AFM)	[73]
	*E. coli*	Mechanical oscillations	Microcantilever and optical displacement transducer	[74]
	*Rhodococcus wratislaviensis*	Cantilever's surface stress	AFM	[75]
	*Listeria monocytogenes*	Presence of bacteria	Infrared radiation excitation, microfluidic cantilever	[76]
	*Chlamydomonas reinhardtii*	Flagellar forces	Micropipette force sensors	[77]

Electrochemical transduction infers biological activity from changes in redox balance, electron transfer or enzymatically coupled electroactive reactions that can be detected using electrodes or redox‐sensitive mediators [78]. Redox reactions underpin energy metabolism, with electrons transferred through ordered pathways such as the electron transport chain. In bioelectronic contexts, these processes can be coupled to an electronic readout, an approach that has been successfully demonstrated in naturally electroactive microorganisms [79]. The ability of yeast to generate electrical outputs from metabolic activity was first demonstrated over a century ago, with early work showing that fermenting *S. cerevisiae* cultures produce measurable electromotive force during the decomposition of organic substrates [65]. More recent efforts to realise this functionality in bioelectronic systems have relied on mediator‐based approaches, with early studies suggesting that *S. cerevisiae* could transfer electrons from cell surface and intracellular redox systems [56, 80]. However, subsequent work indicates that electron transfer is predominantly mediated by flavoproteins within an extracellular polymeric matrix rather than via native extracellular electron transfer pathways [66]. As a result, redox‐based transduction in yeast remains constrained by the absence of dedicated extracellular electron transfer machinery and the disruptive nature of redox perturbation, as altering redox balance directly interferes with cellular metabolism and regulation. Although engineering strategies such as mediator‐based coupling or heterologous electron transfer pathways may partially address these limitations, they typically rely on nonnative components and impose additional physiological burden and therefore remain less mature than comparable approaches in naturally electroactive microorganisms. Furthermore, approaches based on direct electron transfer require close cell–electrode contact, which is difficult to reconcile with suspension cultures, population heterogeneity and industrially relevant operation, limiting scalability and deployment.

Chemical transduction, in this context, refers to the production and secretion of defined molecules that are sampled from the extracellular environment and detected by downstream chemical or electronic sensors. Unlike electrochemical transduction, which often requires direct cell–electrode contact and can disrupt cellular physiology, chemical transduction is generally less invasive and more compatible with scalable and modular systems. In yeast, chemical transduction is often more practical than direct electrochemical coupling and can be engineered to report on internal computational states through regulated metabolite or signal molecule production [62]. However, chemical signals are subject to dilution, temporal delay and interference from complex media, requiring careful design to ensure that measured outputs reflect engineered system states rather than general metabolic activity.

Optical transduction converts biological states into light‐based signals, typically through fluorescent or luminescent reporters, which are then detected by photodiodes, cameras or imaging systems and processed electronically [67]. Although optical signals are not electrical in origin, they form a central component of many bioelectronic workflows due to their noninvasive nature, high sensitivity and compatibility with parallel and spatially resolved measurement. For programmable yeast, optical transduction is particularly attractive, since it enables noninvasive monitoring of internal regulatory states without requiring physical contact. Its primary limitations lie in signal integration at scale, as light scattering and cell shadowing in dense cultures can distort measured outputs, together with constraints imposed by optical accessibility and external instrumentation. Nevertheless, advances in reactor design, optical hardware and computational signal processing offer potential routes to mitigating these limitations.

Physical and mechanical transduction strategies infer the biological state from changes in bulk properties such as impedance [68], dielectric behaviour [69], optical [70] or mechanical characteristics of cell populations [71]. Although these approaches are typically label‐free and minimally invasive, and suited well for population‐level monitoring, they offer limited specificity and are sensitive to environmental and operational variability. As a result, physical transduction is most effective for coarse system‐level measurements or as a complementary modality alongside more specific chemical or optical readouts.

These transduction methods illustrate that biological‐to‐electronic interfacing extends well beyond direct electron extraction from cells. For programmable yeast, the primary challenge is not maximising signal sensitivity, but exposing stabilised, interpretable biological states that are compatible with indirect, scalable detection. This shifts the emphasis of bioelectronics from molecular signal capture towards interface design, setting the stage for electronic control and hybrid feedback systems that integrate biological computation with external decision‐making.

### 4.2 Electronic‐to‐Biological Control

Electronic‐to‐biological control refers to the use of electronic systems to modulate biological behaviour through defined external inputs. Unlike biological‐to‐electronic transduction, which extracts information from living systems, electronic‐to‐biological interfaces enforce control by delivering signals that influence the cellular state. A range of stimuli that can be electronically generated or controlled has been explored, including optical [72], electrical [73], chemical [74], thermal [75] and magnetic [76, 77]. Rather than surveying all stimuli in detail, two promising strategies, namely optogenetic and electrogenetic control, are highlighted to illustrate both the opportunities and constraints of electronic actuation in yeast. Optogenetic systems are well established in yeast and enable reversible control of cellular processes, whereas electrical inputs, although less mature in yeast, offer a direct route for interfacing with electronic systems.

Optogenetic control has been widely adopted in yeast by linking light‐responsive regulatory elements to genetic circuits [72, 80]. Electronic control of illumination enables precise, noninvasive modulation of gene expression with high temporal and spatial resolution [81]. These properties make optogenetics particularly compatible with programmable yeast, allowing electronic systems to trigger regulatory transitions, modulate circuit behaviour or engage memory mechanisms without continuous intervention.

In contrast, direct electrical stimulation is generally poorly suited to yeast, as it lacks dedicated pathways for sensing and transducing electrical signals. Application of electric fields or currents often induces nonspecific effects such as membrane perturbation and stress responses. Electrogenetic approaches attempt to overcome these limitations by linking electrical inputs to engineered genetic regulation. However, reliable implementations in yeast remain challenging [78], underscoring the difficulty of translating electrical signals into controlled biological state changes in nonexcitable systems.

Across these approaches, a common design principle emerges, and effective electronic‐to‐biological control operates through engineered inputs rather than direct physical stimulation. From an engineering perspective, electronic control is therefore best understood as actuation, guiding biological systems through designed regulatory pathways that respond predictably to external commands. This framing aligns electronic control with biological computation and naturally sets the stage for closed‐loop bioelectronic systems integrating sensing, computation and actuation.

### 4.3 Living Sensors and Signal Transduction to Electronics

**FIGURE 3:**
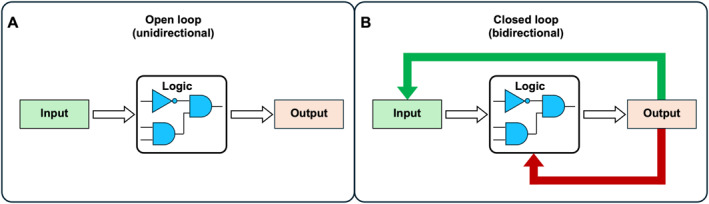
Open‐ and closed‐loop bioelectronic control architectures. (A) Open‐loop (unidirectional) control in which an input is processed through a regulatory or computational module to generate an output without feedback. In this configuration, biological and electronic components operate independently of downstream system states. (B) Closed‐loop (bidirectional) control in which outputs are continuously monitored and fed back to the control logic, enabling the dynamic adjustment of inputs in response to system behaviour and environmental perturbations.

As stated before, bioelectronic interfaces can be broadly sub‐classified as open‐loop or closed‐loop systems, depending on whether feedback needs to be incorporated. In open‐loop (unidirectional) systems, electronic inputs are applied to biological systems or biological outputs, which is then measured without feedback (Figure 3A). In closed‐loop (bidirectional) systems, biological outputs are continuously monitored and used to inform future electronic inputs (assisting with ‘memory’), forming a feedback cycle between biology and electronics [79] (Figure 3B).

Many early bioelectronic interfaces operate under open‐loop conditions. Although suitable for proof‐of‐concept studies and short‐term experiments, unidirectional control is poorly matched to living systems, which are inherently dynamic due to growth, adaptation, stochastic variation and environmental fluctuation. As a result, fixed input–output relationships degrade over time, manifesting as signal drift and reduced interpretability. These effects reflect fundamental properties of living systems rather than experimental noise and become increasingly limiting as the complexity of engineered system increases.

Closed‐loop control offers a fundamentally different and essential model; such systems could enable real‐time monitoring of cellular states and dynamic adjustment of environmental or genetic inputs. By explicitly incorporating feedback, closed‐loop systems can compensate for drift, variability and unanticipated perturbations, maintaining biological states within defined bounds rather than attempting to enforce static behaviours.

A small number of studies have begun to demonstrate what true closed‐loop biological control architectures look like in practice. For example, a cybergenetic platform in which the cell‐cycle state of individual yeast cells was monitored in real time using fluorescence microscopy and a computer‐based controller dynamically adjusted nutrient availability through a microfluidic system to synchronise population‐level cell‐cycle progression. In this system, the biological state of the cells serves as the measured signal, whereas the electronic controller computes corrective inputs that are actuated through automated environmental perturbations, thereby implementing a classical sense–compute–actuate feedback loop [82].

Similar control architectures have also been demonstrated in bioprocess settings. For instance, automated cultivation platforms can monitor intracellular physiological states using fluorescent biosensors measured by flow cytometry and dynamically adjust substrate feeding rates through computer‐controlled pumps. In such systems, biosensor output functions as the feedback signal that informs real‐time adjustments to cultivation conditions, allowing cellular physiology to directly regulate process inputs [83].

These examples illustrate the feasibility of closed‐loop biological control systems; however, relatively few bioelectronic platforms currently implement such strategies, highlighting a persistent gap between conceptual promise and practical realisation. In programmable yeast, closed‐loop control offers a natural framework for integrating engineered genome architectures and regulatory networks with external electronic regulation, allowing cellular states to be monitored and environmental inputs dynamically adjusted in response to measured outputs [59].

## 5 Are We at the Brink of Bioelectronic Yeast?

The convergence of synthetic genomics, biological computation and bioelectronic interfacing prompts an assessment of readiness: To what extent can programmable yeast support reliable and scalable bioelectronic systems? Although the concept of bioelectronic yeast can still encompass both open‐loop and closed‐loop systems, achieving sustained closed‐loop operation represents a critical threshold before system maturity is ultimately achieved. Assessing proximity to this threshold requires an understanding of persistent bottlenecks inherent to living systems and recent enabling advances in genome engineering, standardisation and control infrastructure such as AI.

### 5.1 Bottlenecks

Despite substantial progress in synthetic genomics and biological computation, several fundamental bottlenecks continue to constrain the development of robust bioelectronic yeast systems. Among these include the instability of biological signals over time, the pervasive influence of biological noise and the mismatch between biological and electronic timescales. Each of these challenges reflects intrinsic properties of living systems and becomes increasingly consequential as biological computation is paired with electronic measurement and control. Understanding how these bottlenecks manifest in yeast and how they can be mitigated through design rather than eliminated outright is central to assessing readiness for bioelectronic integration.

#### 5.1.1 Signal Stability

A persistent challenge when interfacing bioelectronic systems is the stability of biological signals over time. Living systems are dynamic by nature, with gene expression, metabolism and population composition continuously evolving in response to internal and external cues [84]. As a result, signals used for electronic readout can drift, saturate or diverge from the intended biological state. This instability is compounded by growth‐associated changes, metabolic reprogramming and adaptive responses to environmental conditions that are intrinsic to living systems and prominent in yeast‐based platforms. Even engineered outputs designed for transduction can vary across time and across populations, limiting their utility for sustained operation. Partial solutions lie in the use of discrete or state‐based outputs, such as memory elements, threshold responses or population‐level averaging, which reduce sensitivity to continuous physiological drift.

#### 5.1.2 Noise

Biological noise arises from stochastic molecular interactions, heterogeneous cellular states and fluctuating environments [84]. Although such variability can be advantageous for adaptation [85], it poses a significant challenge for bioelectronic systems that require interpretable and reproducible signals. In programmable yeast, noise manifests at multiple levels, including transcriptional variability, circuit‐to‐circuit interference and population heterogeneity. When coupled directly to electronic systems, this variability can obscure meaningful state changes and complicate control strategies. Managing noise therefore requires design approaches that operate above the level of individual molecular events, favouring population‐averaged, buffered or memory‐encoded representations of the biological state rather than the raw signal readout.

#### 5.1.3 Timescale Mismatch

One of the most fundamental bottlenecks lies not in the absence of fast biological dynamics but in the mismatch between electronic timescales and those biological processes typically used for engineered computation and control. Although many molecular interactions in cells, such as protein–protein binding or ligand recognition, occur on microsecond to millisecond timescales comparable to electronic systems, the biological states most often exploited for programmable behaviour, including transcription, translation, metabolic reprogramming and population dynamics, unfold over minutes to hours. In practice, near‐real‐time bioelectronic operation therefore depends on selecting biological outputs that report on fast, upstream processes, such as metabolite levels or protein activity, rather than on gene expression alone. For example, metabolite‐based reporters in yeast can reflect changes in the cellular state on timescales of seconds to minutes [86], substantially reducing latency between the biological response and electronic readout.

In addition to technical constraints, biosafety and containment represent a critical bottleneck for the deployment of bioelectronic yeast systems outside controlled laboratory environments. Engineered strains incorporating synthetic chromosomes, orthogonal regulatory systems and externally addressable interfaces may raise regulatory and environmental concerns, particularly where long‐term stability and environmental persistence are uncertain. A range of genetic safeguard strategies has been developed to address these risks, including auxotrophy‐based containment [87], kill‐switch circuits [88] and, more recently, containment approaches that prevent engineered genetic information from functioning outside defined biological contexts [89, 90]. Although these strategies provide promising routes towards improved biocontainment, their integration into complex, programmable yeast platforms remains an active area of research and will be essential for enabling safe and scalable real‐world applications.

### 5.2 Enablers

Despite these constraints, several recent advances have significantly altered the feasibility of bioelectronic yeast systems. Rather than eliminating biological instability, noise or timescale mismatch, these developments have designed around them. Progress in genome‐scale engineering, standardisation and automation, including AI‐assisted workflows, has shifted bioelectronic integration from an exploratory exercise to a tractable engineering problem. Understanding how these enablers reshape the design landscape is essential for assessing whether programmable yeast is approaching practical readiness for bioelectronic systems.

#### 5.2.1 Synthetic Genomes

The completion of fully synthetic yeast chromosomes represents a qualitative shift in how biological systems can be designed and controlled. Synthetic genomes enable structural decisions, such as modular organisation, orthogonal control points and engineered plasticity, to be embedded at the genome scale rather than layered onto evolved structures [14, 26]. This level of control allows biologically meaningful states to be deliberately specified, stabilised and revisited, rather than inferred from fragile, context‐dependent signals.

#### 5.2.2 Standardisation

Standardisation underpins scalability in all engineering disciplines, and synthetic biology is no exception. The development of standardised genetic parts, characterised integration loci, regulatory architectures and formal design languages has made it possible to frame increasingly complex biological systems with predictable behaviour. For example, the Synthetic Biology Open Language (SBOL) model provides a shared, machine‐readable representation of biological components, interactions and system architectures, enabling designs to be specified, exchanged and interpreted across tools and research contexts [91]. In bioelectronics, such standardisation supports consistent mapping between biological states and electronic representations, enabling reproducibility across experiments, devices and platforms.

#### 5.2.3 Automation and AI

As programmable yeast systems and bioelectronic interfaces increase in complexity, the primary challenge shifts from feasibility to coordination, prediction and control. Automation and AI‐driven tools have emerged as enabling infrastructures that accelerate the DBTL cycle [92], support predictive modelling and assist in managing variability across biological and electronic domains. Advances in laboratory robotics, high‐throughput assembly and automated phenotyping now enable the parallel construction and testing of hundreds to thousands of genetic variants, substantially expanding the accessible design space and reducing dependence on manual experimentation.

In programmable yeast, AI methods are increasingly used to explore large design spaces, inform circuit and pathway optimisation and support supervisory control in closed‐loop systems [93], which has been termed as ‘SynBio×AI’. Importantly, automation and AI are synergistic: automated platforms generate the large, structured datasets required for effective model testing, whereas AI‐guided design and analysis prioritise informative experiments and reduce human bottlenecks in decision‐making. Together, these tools do not replace biological insights or design intent but function as layers that help manage noise, drift and timing mismatches by integrating data, prioritising design choices and coordinating control within human‐defined constraints.

## 6 Future Applications of Bioelectronic Yeast

Within this emerging design framework, several classes of future applications become conceivable. Programmable bioelectronic yeast could serve as living sensors and decision‐making layers in bioprocess monitoring, where biological computation filters complex environmental inputs into stable, interpretable states for electronic control. More broadly, such systems may enable hybrid platforms in which yeast populations act as adaptive interfaces between chemical environments and digital infrastructure, supporting applications ranging from environmental sensing and diagnostics to smart biomanufacturing and adaptive control systems. Importantly, these applications do not rely on continuous, high‐resolution molecular readout but instead exploit engineered state transitions, memory and population‐level averaging to produce robust, system‐level signals compatible with electronic integration.

The fundamental limitations imposed by biology have not disappeared. Rather, advances in genome‐scale engineering, standardisation and control infrastructure have made it possible to design around them in systematic ways. Although the concept of true bioelectronic yeast models are not yet a plug‐and‐play technology, the field has moved beyond speculative vision towards a coherent design framework in which progress can be measured, reproduced and iteratively improved. The ‘brink’ is therefore not defined by technological perfection but by the emergence of engineering principles that will make biological–electronic integration a solvable problem rather than an aspirational one.

## Author Contributions

Paige E. Erpf, Bill D. Kinder, Roy S. K. Walker, Edward Archer, Ian T. Paulsen, and Isak S. Pretorius conceived, developed, and edited the manuscript.

## Funding

This article forms part of Macquarie University's Bioinnovation Initiative. The Sc2.0 component of this initiative is financially supported by Macquarie University, Bioplatforms of Australia, the New South Wales (NSW) Chief Scientist and Engineering and the NSW Government's Department of Primary Industries. Australian Government funding through its investment agency, the Australian Research Council, towards the Macquarie University‐led ARC Centre of Excellence in Synthetic Biology is gratefully acknowledged.

## Conflicts of Interest

The authors declare no conflicts of interest.

## Data Availability

Data sharing not applicable ‐ no new data generated, or the article describes entirely theoretical research.

## References

[1] I. S. Pretorius , “Visualizing the Next Frontiers in Wine Yeast Research,” FEMS Yeast Research 22, no. 1 (2022): foac010, 10.1093/femsyr/foac010. 35175339 PMC8916113

[2] P. J. Chambers and I. S. Pretorius , “Fermenting Knowledge: The History of Winemaking, Science and Yeast Research,” EMBO Reports 11, no. 12 (2010): 914–920, 10.1038/embor.2010.179. 21072064 PMC2999870

[3] J. Steensels , B. Gallone , K. Voordeckers , and K. J. Verstrepen , “Domestication of Industrial Microbes,” Current Biology 29, no. 10 (2019): R381–R393, 10.1016/j.cub.2019.04.025. 31112692

[4] T. A. Dixon and I. S. Pretorius , “Drawing on the Past to Shape the Future of Synthetic Yeast Research,” International Journal of Molecular Sciences 21, no. 19 (2020): 7156, 10.3390/ijms21197156. 32998303 PMC7583028

[5] J. Nielsen , “The Power of Yeast,” Yeast 42, no. 12 (2025): 303–310, 10.1002/yea.70009. 41445307 PMC12757823

[6] D. Botstein and G. R. Fink , “Yeast: An Experimental Organism for 21st Century Biology,” Genetics 189, no. 3 (2011): 695–704, 10.1534/genetics.111.130765. 22084421 PMC3213361

[7] A. Goffeau , B. G. Barrell , H. Bussey , et al., “Life With 6000 Genes,” Science 274, no. 5287 (1996): 546–567, 63–67, 10.1126/science.274.5287.546. 8849441

[8] S. G. Oliver , “From DNA Sequence to Biological Function,” Nature 379, no. 6566 (1996): 597–600, 10.1038/379597a0. 8628394

[9] S. G. Oliver , “Functional Genomics: Lessons From Yeast,” Philosophical Transactions of the Royal Society of London B Biological Sciences 357, no. 1417 (2002): 17–23, 10.1098/rstb.2001.1049. 11839178 PMC1692912

[10] S. G. Oliver , “Yeast Systems Biology: The Continuing Challenge of Eukaryotic Complexity,” Methods in Molecular Biology 2049 (2019): 3–13, 10.1007/978-1-4939-9736-7_1. 31602602

[11] S. Shi , Y. Chen , and J. Nielsen , “Metabolic Engineering of Yeast,” Annual Review of Biophysics 54, no. 1 (2025): 101–120, 10.1146/annurev-biophys-070924-103134. 39836878

[12] M. Heinemann and S. Panke , “Synthetic Biology–Putting Engineering Into Biology,” Bioinformatics 22, no. 22 (2006): 2790–2799, 10.1093/bioinformatics/btl469. 16954140

[13] I. S. Pretorius , T. A. Dixon , M. Boers , I. T. Paulsen , and D. L. Johnson , “The Coming Wave of Confluent Biosynthetic, Bioinformational and Bioengineering Technologies,” Nature Communications 16, no. 1 (2025): 2959, 10.1038/s41467-025-58030-y. PMC11947079 40140397

[14] S. M. Richardson , L. A. Mitchell , G. Stracquadanio , et al., “Design of a Synthetic Yeast Genome,” Science 355, no. 6329 (2017): 1040–1044, 10.1126/science.aaf4557. 28280199

[15] P. E. Erpf , F. Meier , R. S. K. Walker , et al., “Building Synthetic Chromosomes One Yeast at a Time: Insights From Sc2.0,” Nature Biotechnology 43, no. 12 (2025): 1911–1918, 10.1038/s41587-025-02913-4. 41381913

[16] H. D. Goold , H. Kroukamp , P. E. Erpf , et al., “Construction and Iterative Redesign of synXVI a 903 kb Synthetic *Saccharomyces cerevisiae* Chromosome,” Nature Communications 16, no. 1 (2025): 841, 10.1038/s41467-024-55318-3. PMC11747415 39833175

[17] T. C. Williams , H. Kroukamp , X. Xu , et al., “Parallel Laboratory Evolution and Rational Debugging Reveal Genomic Plasticity to *S. cerevisiae* Synthetic Chromosome XIV Defects,” Cell Genomics 3, no. 11 (2023): 100379, 10.1016/j.xgen.2023.100379. 38020977 PMC10667330

[18] X. L. Wu , B. Z. Li , W. Z. Zhang , et al., “Genome‐Wide Landscape of Position Effects on Heterogeneous Gene Expression in *Saccharomyces cerevisiae* ,” Biotechnology for Biofuels 10, no. 1 (2017): 189, 10.1186/s13068-017-0872-3. 28729884 PMC5516366

[19] D. B. Flagfeldt , V. Siewers , L. Huang , and J. Nielsen , “Characterization of Chromosomal Integration Sites for Heterologous Gene Expression in *Saccharomyces cerevisiae* ,” Yeast 26, no. 10 (2009): 545–551, 10.1002/yea.1705. 19681174

[20] A. Becskei , B. B. Kaufmann , and A. van Oudenaarden , “Contributions of Low Molecule Number and Chromosomal Positioning to Stochastic Gene Expression,” Nature Genetics 37, no. 9 (2005): 937–944, 10.1038/ng1616. 16086016

[21] E. Fajiculay and C. P. Hsu , “Localization of Noise in Biochemical Networks,” ACS Omega 8, no. 3 (2023): 3043–3056, 10.1021/acsomega.2c06113. 36713703 PMC9878546

[22] M. D. Mikkelsen , L. D. Buron , B. Salomonsen , et al., “Microbial Production of Indolylglucosinolate Through Engineering of a Multi‐Gene Pathway in a Versatile Yeast Expression Platform,” Metabolic Engineering 14, no. 2 (2012): 104–111, 10.1016/j.ymben.2012.01.006. 22326477

[23] M. Babaei , L. Sartori , A. Karpukhin , D. Abashkin , E. Matrosova , and I. Borodina , “Expansion of EasyClone‐MarkerFree Toolkit for *Saccharomyces cerevisiae* Genome With New Integration Sites,” FEMS Yeast Research 21, no. 4 (2021): foab027, 10.1093/femsyr/foab027. 33893795 PMC8112480

[24] M. M. Jessop‐Fabre , T. Jakociunas , V. Stovicek , et al., “EasyClone‐MarkerFree: A Vector Toolkit for Marker‐Less Integration of Genes Into *Saccharomyces cerevisiae* via CRISPR‐Cas9,” Biotechnology Journal 11, no. 8 (2016): 1110–1117, 10.1002/biot.201600147. 27166612 PMC5094547

[25] D. Schindler , R. S. K. Walker , S. Jiang , et al., “Design, Construction, and Functional Characterization of a tRNA Neochromosome in Yeast,” Cell 186, no. 24 (2023): 5237–5253.e22, 10.1016/j.cell.2023.10.015. 37944512

[26] J. S. Dymond , S. M. Richardson , C. E. Coombes , et al., “Synthetic Chromosome Arms Function in Yeast and Generate Phenotypic Diversity by Design,” Nature 477, no. 7365 (2011): 471–476, 10.1038/nature10403. 21918511 PMC3774833

[27] N. G. Kuijpers , D. Solis‐Escalante , M. A. Luttik , et al., “Pathway Swapping: Toward Modular Engineering of Essential Cellular Processes,” Proceedings of the National Academy of Sciences of the United States of America 113, no. 52 (2016): 15060–15065, 10.1073/pnas.1606701113. 27956602 PMC5206561

[28] M. Tominaga , A. Kondo , and J. Ishii , “Engineering of Synthetic Transcriptional Switches in Yeast,” Life (Basel) 12, no. 4 (2022): 557, 10.3390/life12040557. 35455048 PMC9030632

[29] M. Tominaga , Y. Shima , K. Nozaki , et al., “Designing Strong Inducible Synthetic Promoters in Yeasts,” Nature Communications 15, no. 1 (2024): 10653, 10.1038/s41467-024-54865-z. PMC11659477 39702268

[30] L. Nguyen , B. Schmelzer , S. Wilkinson , and D. Mattanovich , “From Natural to Synthetic: Promoter Engineering in Yeast Expression Systems,” Biotechnology Advances 77 (2024): 108446, 10.1016/j.biotechadv.2024.108446. 39245291

[31] T. Y. Chiu and J. R. Jiang , “Logic Synthesis of Recombinase‐Based Genetic Circuits,” Scientific Reports 7, no. 1 (2017): 12873, 10.1038/s41598-017-07386-3. 28993615 PMC5634492

[32] R. A. Ashraf , M. Bureik , and M. A. Marchisio , “Design and Engineering of Logic Genetic‐Enzymatic Gates Based on the Activity of the Human CYP2C9 Enzyme in Permeabilized *Saccharomyces cerevisiae* Cells,” Synthetic and Systems Biotechnology 9, no. 3 (2024): 406–415, 10.1016/j.synbio.2024.03.013. 38590712 PMC10999488

[33] M. M. Müller , K. M. Arndt , and S. A. Hoffmann , “Genetic Circuits in Synthetic Biology: Broadening the Toolbox of Regulatory Devices,” Frontiers in Synthetic Biology 3 (2025): 1548572, 10.3389/fsybi.2025.1548572.

[34] Q. Wang and L. Wang , “Genetic Incorporation of Unnatural Amino Acids Into Proteins in Yeast,” Methods in Molecular Biology 794 (2012): 199–213, 10.1007/978-1-61779-331-8_12. 21956564 PMC3965369

[35] D. A. Bushnell , P. Cramer , and R. D. Kornberg , “Selenomethionine Incorporation in *Saccharomyces cerevisiae* RNA Polymerase II,” Structure 9, no. 1 (2001): R11–R14, 10.1016/s0969-2126(00)00554-2. 11342141

[36] T. Xu , C. A. Johnson , J. E. Gestwicki , and A. Kumar , “Conditionally Controlling Nuclear Trafficking in Yeast by Chemical‐Induced Protein Dimerization,” Nature Protocols 5, no. 11 (2010): 1831–1843, 10.1038/nprot.2010.141. 21030958 PMC4976631

[37] K. H. Siu and W. Chen , “Control of the Yeast Mating Pathway by Reconstitution of Functional Alpha‐Factor Using Split Intein‐Catalyzed Reactions,” ACS Synthetic Biology 6, no. 8 (2017): 1453–1460, 10.1021/acssynbio.7b00078. 28505429

[38] T. S. Gardner , C. R. Cantor , and J. J. Collins , “Construction of a Genetic Toggle Switch in *Escherichia coli* ,” Nature 403, no. 6767 (2000): 339–342, 10.1038/35002131. 10659857

[39] M. B. Elowitz and S. Leibler , “A Synthetic Oscillatory Network of Transcriptional Regulators,” Nature 403, no. 6767 (2000): 335–338, 10.1038/35002125. 10659856

[40] A. A. Nielsen and C. A. Voigt , “Multi‐Input CRISPR/Cas Genetic Circuits That Interface Host Regulatory Networks,” Molecular Systems Biology 10, no. 11 (2014): 763, 10.15252/msb.20145735. 25422271 PMC4299604

[41] C. J. Bashor , N. C. Helman , S. Yan , and W. A. Lim , “Using Engineered Scaffold Interactions to Reshape MAP Kinase Pathway Signaling Dynamics,” Science 319, no. 5869 (2008): 1539–1543, 10.1126/science.1151153. 18339942

[42] A. S. Khalil , T. K. Lu , C. J. Bashor , et al., “A Synthetic Biology Framework for Programming Eukaryotic Transcription Functions,” Cell 150, no. 3 (2012): 647–658, 10.1016/j.cell.2012.05.045. 22863014 PMC3653585

[43] Z. Liu , Y. Zhang , and J. Nielsen , “Synthetic Biology of Yeast,” Biochemistry 58, no. 11 (2019): 1511–1520, 10.1021/acs.biochem.8b01236. 30618248

[44] W. S. Teo and M. W. Chang , “Development and Characterization of AND‐Gate Dynamic Controllers With a Modular Synthetic GAL1 Core Promoter in *Saccharomyces cerevisiae* ,” Biotechnology and Bioengineering 111, no. 1 (2014): 144–151, 10.1002/bit.25001. 23860786

[45] A. Rantasalo , J. Kuivanen , M. Penttila , J. Jantti , and D. Mojzita , “Synthetic Toolkit for Complex Genetic Circuit Engineering in *Saccharomyces cerevisiae* ,” ACS Synthetic Biology 7, no. 6 (2018): 1573–1587, 10.1021/acssynbio.8b00076. 29750501 PMC6150731

[46] S. Castano‐Cerezo , M. Fournie , P. Urban , J. L. Faulon , and G. Truan , “Development of a Biosensor for Detection of Benzoic Acid Derivatives in *Saccharomyces cerevisiae* ,” Frontiers in Bioengineering and Biotechnology 7 (2019): 372, 10.3389/fbioe.2019.00372. 31970152 PMC6959289

[47] K. V. Presnell , O. Melhem , N. J. Morse , and H. S. Alper , “Modular, Synthetic Boolean Logic Gates Enabled in *Saccharomyces cerevisiae* Through T7 Polymerases/CRISPR dCas9 Designs,” ACS Synthetic Biology 11, no. 10 (2022): 3414–3425, 10.1021/acssynbio.2c00327. 36206523

[48] M. Acar , A. Becskei , and A. van Oudenaarden , “Enhancement of Cellular Memory by Reducing Stochastic Transitions,” Nature 435, no. 7039 (2005): 228–232, 10.1038/nature03524. 15889097

[49] C. M. Ajo‐Franklin , D. A. Drubin , J. A. Eskin , et al., “Rational Design of Memory in Eukaryotic Cells,” Genes & Development 21, no. 18 (2007): 2271–2276, 10.1101/gad.1586107. 17875664 PMC1973140

[50] D. G. Brickner , I. Cajigas , Y. Fondufe‐Mittendorf , et al., “H2A.Z‐Mediated Localization of Genes at the Nuclear Periphery Confers Epigenetic Memory of Previous Transcriptional State,” PLoS Biology 5, no. 4 (2007): e81, 10.1371/journal.pbio.0050081. 17373856 PMC1828143

[51] B. Sump and J. Brickner , “Establishment and Inheritance of Epigenetic Transcriptional Memory,” Frontiers in Molecular Biosciences 9 (2022): 977653, 10.3389/fmolb.2022.977653. 36120540 PMC9479176

[52] I. Belda , T. C. Williams , M. de Celis , I. T. Paulsen , and I. S. Pretorius , “Seeding the Idea of Encapsulating a Representative Synthetic Metagenome in a Single Yeast Cell,” Nature Communications 12, no. 1 (2021): 1599, 10.1038/s41467-021-21877-y. PMC7952416 33707418

[53] A. Tamsir , J. J. Tabor , and C. A. Voigt , “Robust Multicellular Computing Using Genetically Encoded NOR Gates and Chemical ‘Wires’,” Nature 469, no. 7329 (2011): 212–215, 10.1038/nature09565. 21150903 PMC3904220

[54] S. Regot , J. Macia , N. Conde , et al., “Distributed Biological Computation With Multicellular Engineered Networks,” Nature 469, no. 7329 (2011): 207–211, 10.1038/nature09679. 21150900

[55] R. S. K. Walker and I. S. Pretorius , “Synthetic Biology for the Engineering of Complex Wine Yeast Communities,” Nature Food 3, no. 4 (2022): 249–254, 10.1038/s43016-022-00487-x. 37118192

[56] L. Li , Y. Pan , S. Zhang , et al., “Quorum Sensing: Cell‐to‐Cell Communication in *Saccharomyces cerevisiae* ,” Frontiers in Microbiology 14 (2023): 1250151, 10.3389/fmicb.2023.1250151. 38075875 PMC10701894

[57] U. Gutbier , J. Korp , L. Scheufler , and K. Ostermann , “Genetic Modules for Alpha‐Factor Pheromone Controlled Growth Regulation of *Saccharomyces cerevisiae* ,” Engineering in Life Sciences 24, no. 8 (2024): e2300235, 10.1002/elsc.202300235. 39113811 PMC11300815

[58] Y. C. Heng , S. Kitano , A. V. Susanto , J. L. Foo , and M. W. Chang , “Tunable Cell Differentiation via Reprogrammed Mating‐Type Switching,” Nature Communications 15, no. 1 (2024): 8163, 10.1038/s41467-024-52282-w. PMC11408693 39289346

[59] J. Rivnay , R. Raman , J. T. Robinson , et al., “Integrating Bioelectronics With Cell‐Based Synthetic Biology,” Nature Reviews Bioengineering 3, no. 4 (2025): 317–332, 10.1038/s44222-024-00262-6.

[60] F. J. Rawson , A. J. Gross , D. J. Garrett , A. J. Downard , and K. H. R. Baronian , “Mediated Electrochemical Detection of Electron Transfer From the Outer Surface of the Cell Wall of *Saccharomyces cerevisiae* ,” Electrochemistry Communications 15, no. 1 (2012): 85–87, 10.1016/j.elecom.2011.11.030.

[61] F. J. Rawson , A. J. Downard , and K. H. Baronian , “Electrochemical Detection of Intracellular and Cell Membrane Redox Systems in *Saccharomyces cerevisiae* ,” Scientific Reports 4, no. 1 (2014): 5216, 10.1038/srep05216. 24910017 PMC4048887

[62] F. Monteiro , G. Hubmann , V. Takhaveev , et al., “Measuring Glycolytic Flux in Single Yeast Cells With an Orthogonal Synthetic Biosensor,” Molecular Systems Biology 15, no. 12 (2019): e9071, 10.15252/msb.20199071. 31885198 PMC6920703

[63] M. Guenther , F. Altenkirch , K. Ostermann , et al., “Optical and Impedimetric Study of Genetically Modified Cells for Diclofenac Sensing,” Journal of Sensors and Sensor Systems 8, no. 1 (2019): 215–222, 10.5194/jsss-8-215-2019.

[64] P. J. Snyder , D. R. LaJeunesse , P. Reddy , R. Kirste , R. Collazo , and A. Ivanisevic , “Bioelectronics Communication: Encoding Yeast Regulatory Responses Using Nanostructured Gallium Nitride Thin Films,” Nanoscale 10, no. 24 (2018): 11506–11516, 10.1039/c8nr03684e. 29888776 PMC6195121

[65] M. C. Potters , “Electrical Effects Accompanying the Decomposition of Organic Compounds,” Proceedings of the Royal Society of London ‐ Series B: Containing Papers of a Biological Character 84, no. 571 (1911): 260–276, 10.1098/rspb.1911.0073.

[66] G. C. Sedenho , I. Modenez , G. R. Mendes , and F. N. Crespilho , “The Role of Extracellular Polymeric Substance Matrix on *Saccharomyces cerevisiae* Bioelectricity,” Electrochimica Acta 393 (2021): 139080, 10.1016/j.electacta.2021.139080.

[67] S. Assawajaruwan , F. Kuon , M. Funke , and B. Hitzmann , “Feedback Control Based on NADH Fluorescence Intensity for *Saccharomyces cerevisiae* Cultivations,” Bioresources and Bioprocessing 5, no. 1 (2018): 24, 10.1186/s40643-018-0210-z.

[68] S. Abasi , J. R. Aggas , G. G. Garayar‐Leyva , B. K. Walther , and A. Guiseppi‐Elie , “Bioelectrical Impedance Spectroscopy for Monitoring Mammalian Cells and Tissues Under Different Frequency Domains: A Review,” ACS Measurement Science Au 2, no. 6 (2022): 495–516, 10.1021/acsmeasuresciau.2c00033. 36785772 PMC9886004

[69] G. Flores‐Cosio , E. J. Herrera‐Lopez , M. Arellano‐Plaza , A. Gschaedler‐Mathis , M. Kirchmayr , and L. Amaya‐Delgado , “Application of Dielectric Spectroscopy to Unravel the Physiological State of Microorganisms: Current State, Prospects and Limits,” Applied Microbiology and Biotechnology 104, no. 14 (2020): 6101–6113, 10.1007/s00253-020-10677-x. 32440707

[70] T. Duque , A. C. Chaves Ribeiro , H. S. de Camargo , P. A. Costa Filho , H. P. Mesquita Cavalcante , and D. Lopes , “New Insights on Optical Biosensors: Techniques, Construction and Application,” in State of the Art in Biosensors ‐ General Aspects (IntechOpen, 2013), https://www.intechopen.com/chapters/43447.

[71] P. A. Janmey and C. A. McCulloch , “Cell Mechanics: Integrating Cell Responses to Mechanical Stimuli,” Annual Review of Biomedical Engineering 9 (2007): 1–34, 10.1146/annurev.bioeng.9.060906.151927. 17461730

[72] J. Jang and J. L. Avalos , “Lighting Up Yeast: Overview of Optogenetics in Yeast and Their Applications to Yeast Biotechnology,” FEMS Yeast Research 25 (2025): foaf064, 10.1093/femsyr/foaf064. 41124034 PMC12648543

[73] A. Haupt , A. Campetelli , D. Bonazzi , M. Piel , F. Chang , and N. Minc , “Electrochemical Regulation of Budding Yeast Polarity,” PLoS Biology 12, no. 12 (2014): e1002029, 10.1371/journal.pbio.1002029. 25548923 PMC4280105

[74] A. Nakamura , Y. Goto , H. Sugiyama , S. Tsukiji , and K. Aoki , “Chemogenetic Manipulation of Endogenous Proteins in Fission Yeast Using a Self‐Localizing Ligand‐Induced Protein Translocation System,” ACS Chemical Biology 18, no. 12 (2023): 2506–2515, 10.1021/acschembio.3c00478. 37990966

[75] P. Zhou , W. Xie , Z. Yao , Y. Zhu , L. Ye , and H. Yu , “Development of a Temperature‐Responsive Yeast Cell Factory Using Engineered Gal4 as a Protein Switch,” Biotechnology and Bioengineering 115, no. 5 (2018): 1321–1330, 10.1002/bit.26544. 29315481

[76] K. Nishida and P. A. Silver , “Induction of Biogenic Magnetization and Redox Control by a Component of the Target of Rapamycin Complex 1 Signaling Pathway,” PLoS Biology 10, no. 2 (2012): e1001269, 10.1371/journal.pbio.1001269. 22389629 PMC3289596

[77] M. Adhikari , L. Wang , D. Adhikari , et al., “Electric Stimulation: A Versatile Manipulation Technique Mediated Microbial Applications,” Bioprocess and Biosystems Engineering 48, no. 2 (2025): 171–192, 10.1007/s00449-024-03107-z. 39611964

[78] M. Mansouri and M. Fussenegger , “Engineering Electrogenetic Interfaces for Mammalian Cell Control,” Cell Chemical Biology 32, no. 4 (2025): 521–528, 10.1016/j.chembiol.2025.01.003. 39879984

[79] J. B. Hosseini , K. Zlobina , G. Marquez , et al., “A Feedback Control Architecture for Bioelectronic Devices With Applications to Wound Healing,” Journal of the Royal Society, Interface 18, no. 185 (2021): 20210497, 10.1098/rsif.2021.0497. 34847791 PMC8633799

[80] A. L. A. Perez , L. C. Piva , J. P. C. Fulber , et al., “Optogenetic Strategies for the Control of Gene Expression in Yeasts,” Biotechnology Advances 54 (2022): 107839, 10.1016/j.biotechadv.2021.107839. 34592347

[81] M. Le Bec , S. Pouzet , C. Cordier , et al., “Optogenetic Spatial Patterning of Cooperation in Yeast Populations,” Nature Communications 15, no. 1 (2024): 75, 10.1038/s41467-023-44379-5. PMC10761962 38168087

[82] G. Perrino , S. Napolitano , F. Galdi , et al., “Automatic Synchronisation of the Cell Cycle in Budding Yeast Through Closed‐Loop Feedback Control,” Nature Communications 12, no. 1 (2021): 2452, 10.1038/s41467-021-22689-w. PMC8079375 33907191

[83] R. P. Foncillas , S. Magnusson , B. Al‐Rudainy , O. Wallberg , M. F. Gorwa‐Grauslund , and M. Carlquist , “Automated Yeast Cultivation Control Using a Biosensor and Flow Cytometry,” Journal of Industrial Microbiology & Biotechnology 51 (2024): kuae039, 10.1093/jimb/kuae039. 39424604 PMC11561399

[84] M. B. Elowitz , A. J. Levine , E. D. Siggia , and P. S. Swain , “Stochastic Gene Expression in a Single Cell,” Science 297, no. 5584 (2002): 1183–1186, 10.1126/science.1070919. 12183631

[85] M. Kaern , T. C. Elston , W. J. Blake , and J. J. Collins , “Stochasticity in Gene Expression: From Theories to Phenotypes,” Nature Reviews Genetics 6, no. 6 (2005): 451–464, 10.1038/nrg1615. 15883588

[86] D. Botman , T. G. O'Toole , J. Goedhart , F. J. Bruggeman , J. H. van Heerden , and B. Teusink , “A Yeast FRET Biosensor Enlightens cAMP Signaling,” Molecular Biology of the Cell 32, no. 13 (2021): 1229–1240, 10.1091/mbc.E20-05-0319. 33881352 PMC8351543

[87] T. Chang , W. Ding , S. Yan , et al., “A Robust Yeast Biocontainment System With Two‐Layered Regulation Switch Dependent on Unnatural Amino Acid,” Nature Communications 14, no. 1 (2023): 6487, 10.1038/s41467-023-42358-4. PMC10576815 37838746

[88] C. Maneira , S. Becker , A. Chamas , and G. Lackner , “A Multilayered Biocontainment System for Laboratory and Probiotic Yeast,” Metabolic Engineering 91 (2025): 442–454, 10.1016/j.ymben.2025.06.009. 40578793

[89] M. Schmidt , “Xenobiology: A New Form of Life as the Ultimate Biosafety Tool,” BioEssays 32, no. 4 (2010): 322–331, 10.1002/bies.200900147. 20217844 PMC2909387

[90] G. Pavão , I. Sfalcin , and D. Bonatto , “Biocontainment Techniques and Applications for Yeast Biotechnology,” Fermentation 9, no. 4 (2023): 341, 10.3390/fermentation9040341.

[91] J. A. McLaughlin , J. Beal , G. Misirli , et al., “The Synthetic Biology Open Language (SBOL) Version 3: Simplified Data Exchange for Bioengineering,” Frontiers in Bioengineering and Biotechnology 8 (2020): 1009, 10.3389/fbioe.2020.01009. 33015004 PMC7516281

[92] N. Gurdo , D. C. Volke , D. McCloskey , and P. I. Nikel , “Automating the Design‐Build‐Test‐Learn Cycle Towards Next‐Generation Bacterial Cell Factories,” New Biotechnology 74 (2023): 1–15, 10.1016/j.nbt.2023.01.002. 36736693

[93] A. Coutant , K. Roper , D. Trejo‐Banos , et al., “Closed‐Loop Cycles of Experiment Design, Execution, and Learning Accelerate Systems Biology Model Development in Yeast,” Proceedings of the National Academy of Sciences of the United States of America 116, no. 36 (2019): 18142–18147, 10.1073/pnas.1900548116. 31420515 PMC6731661

